# The Mediating Role of Psychological Need Thwarting in the Relationship Between Compulsory Citizenship Behavior and Psychological Withdrawal

**DOI:** 10.3389/fpsyg.2019.02595

**Published:** 2019-11-26

**Authors:** Mohsin Bashir, Kanwal Shaheen, Sharjeel Saleem, Mohammed Khurrum Bhutta, Muhammad Abrar, Zhao Jun

**Affiliations:** ^1^Lyallpur Business School, Government College University Faisalabad, Faisalabad, Pakistan; ^2^Department of Business Administration, Government College Women University Faisalabad, Faisalabad, Pakistan; ^3^College of Business, Ohio University, Athens, OH, United States; ^4^School of Public Administration, Zhongnan University of Economics and Law, Wuhan, China

**Keywords:** compulsory citizenship behavior, autonomy need thwarting, relatedness need thwarting, psychological withdrawal, basic psychological needs theory

## Abstract

This study examined a novel process underlying the relationship between compulsory citizenship behavior and psychological withdrawal. Specifically, based on basic psychological needs theory, thwarting of autonomy and relatedness needs were expected to explain the association between compulsory citizenship behavior and psychological withdrawal. The PROCESS macro was used to analyze the data collected from 368 clerical staff working in public universities in Pakistan. The results confirmed that thwarting of autonomy and relatedness need mediated the relationship between compulsory citizenship behavior and psychological withdrawal. This study makes a significant contribution to the unexplored domain of the process employees use to cope with compulsory citizenship behavior. It also highlights the role of an understudied construct, i.e., psychological need thwarting as a crucial motivational mechanism for elucidating the relationship between compulsory citizenship behavior and psychological withdrawal. The findings of this study provide recommendations for future researchers, along with its implications for practitioners.

## Introduction

Compulsory citizenship behavior (CCB hereinafter) is “employees’ engagement in extra-role, but not necessary voluntary, behaviors that are conducted under duress and not as a result of the self-driven goodwill of the individual himself/herself” (Vigoda-Gadot, 2007, p. 387). It has been considered as a work stressor (He et al., 2019), the prevalence of which has been confirmed by different studies in different organizational cultures and work settings (see Zhao et al., 2013; He et al., 2019; Liu et al., 2019). The victimized employees of CCB demonstrate various harmful attitudes and coping behaviors, e.g., turnover intention (Ahmadian et al., 2017), moral disengagement and silence (He et al., 2019), psychological withdrawal (PWd) (Shaheen et al., 2019), etc. However, there is still a need to investigate the mechanisms underlying the relationship between CCB and aversive coping behaviors such as PWd (Shaheen et al., 2019).

Psychological withdrawal is a behavior in which employees remain physically present at work, but they mentally escape from it (Lehman and Simpson, 1992). Such less severe withdrawal behaviors are costly for organizations (Siebert and Zubanov, 2009; Rurkkhum, 2018), as they lead employees to engage in severe withdrawal behaviors, i.e., increased turnover intention (Rurkkhum, 2018), and turnover (Berry et al., 2012). Previous research has highlighted insufficient attention of researchers to work withdrawal behaviors (Wang and Wang, 2017) and emphasized the importance of conducting more studies on exploring its antecedents (Rurkkhum, 2018). So to gain an understanding of how to motivate employees to devote their attention toward good job performance, it is crucial to focus on antecedents of psychological withdrawal such as psychological need thwarting so that they could be controlled.

The current study has the following goals. First, we aim to test psychological need thwarting as an antecedent to coping behavior of PWd by utilizing basic psychological needs theory (BPNT; Ryan and Deci, 2000). The theory postulates that humans have three innate psychological needs: i.e., the need for competence, relatedness, and autonomy. The theory further characterizes that thwarting of these needs leads employees to cope by adopting diverse maladaptive coping behaviors (see Vansteenkiste et al., 2010; Schultz et al., 2015; Olafsen et al., 2017). Thus, the present study tests whether thwarting of psychological needs may serve as an antecedent to maladaptive coping behavior of PWd. Second, the study aims to test whether CCB (a coercive and controlling stressor) may thwart employee psychological needs, as research has shown that controlling/coercive events at workplace thwart psychological needs of employees (e.g., Liu et al., 2017; Bartholomew et al., 2018). Third, the study aims to test whether thwarting of psychological needs serves as a mediational mechanism through which CCB stressor relates to employee PWd behavior. Previously, psychological need thwarting served as a motivational mechanism in explaining the relation of stressors with maladaptive coping behaviors of individuals (see Gillet et al., 2015; Hein et al., 2015; Jang et al., 2016; Olafsen et al., 2017). Hence, CCB may be regarded as a demotivating factor that may thwart employees’ psychological needs and allow them to cope by psychologically withdrawing from work. This study may contribute to the advancement of BPNT, CCB and work withdrawal literature. Along with that, it may be of practical significance in these areas.

Another important contribution of the study is its population of clerical staff working in four large Pakistani public universities located in Faisalabad city. This population was preferred due to the following reasons. First, the work that clerical staff performs is very stressful (Peeters et al., 1995; Crompton, 2011). Second, this human force from university premises has been given less importance in available research on universities (Szekeres, 2006). Lastly, they are perceived to be more vulnerable to CCB due to having low authority and social support at work (e.g., Tsai and Liu, 2012; Rodwell et al., 2013).

In the following sections, we detail the probable positive relationship between CCB and psychological need thwarting. We further continue by explaining the possible positive relationship between psychological need thwarting and PWd. Lastly, the study elaborates on the potential mediating role of psychological need thwarting in relationship between CCB and PWd. We then present the methodology and results of our research and further discuss its theoretical and practical implications, followed by conclusion.

## Theoretical Background and Hypotheses

### CCB and Thwarting of Psychological Needs

Basic psychological needs theory (Ryan and Deci, 2000) identifies three innate human psychological needs, i.e., the need for competence, relatedness, and autonomy. The need for competence refers to an inherent inclination of individuals to feel useful in their continuous interaction with the social environment and have opportunities for the expression of their abilities (White, 1959; Deci and Ryan, 2000). The need for relatedness is an inherent propensity of individuals to feel cared for by people in their social settings (Baumeister and Leary, 1995). The need for autonomy reflects the innate desire of individuals to experience a general sense of choice and volition in a display of their own behavior (DeCharms, 1968; Deci and Ryan, 2000), rather than feeling pressured and coerced. An empirically positive relationship of psychological need satisfaction has been established with persons’ well-being (e.g., Gunnell et al., 2013). On the other hand, the thwarting of these needs was proposed to result in non-optimal development and ill-health of persons (Deci and Ryan, 2000). Psychological need thwarting is an understudied component of self-determination theory (SDT) (Costa et al., 2015). It is considered as a feeling that arises in response to an individual’s perception that his/her psychological needs are actively undermined by others (Bartholomew et al., 2011a). Multiple factors have been explored in past research that are related to psychological need thwarting, e.g., controlling behaviors (Mabbe et al., 2018), role conflict (Gillet et al., 2015), etc. Similarly, we expect that CCB is likely to be associated with autonomy, and relatedness need thwarting due to following reasons:

Previous research established that there are autonomy-supportive contexts (e.g., Chiniara and Bentein, 2016). On the other hand, there are coercive or pressurizing social partners or social contexts as well (e.g., Hein et al., 2015) that have a detrimental effect on psychological needs (e.g., Blanchard et al., 2009). In general, work-life, social partners are expected to provide employees with social support as a social resource to experience need satisfaction. But in the case of CCB at the workplace, social partners are perceived as enforcing employees to perform citizenship behavior against their choice, which breaks the social tradition for support and care from them. This may result in thwarting of relatedness need. The pressure employees feel to fulfill those OCB demands, which they didn’t initiate nor desired may influence their sense of willingness and may thus thwart their autonomy need. Hence, we argue that performance of a citizenship behavior out of pressure from “significant others” against one’s choice might be construed as a lack of consideration of one’s basic psychological needs of autonomy and relatedness. This allows us to hypothesize that

*Hypothesis 1a*. CCB is positively related to thwarting of psychological need for autonomy.
*Hypothesis 1b.* CCB is positively related to thwarting of psychological need for relatedness.

### Psychological Need Thwarting and PWd

Previous research has established a positive relationship between need thwarting and non-optimal functioning of individuals (Trepanier et al., 2015). These results are consistent with SDT, which suggests that if individuals experience psychological need thwarting, there is more likelihood that they will sense a lack of control, helplessness, and alienation (Ntoumanis et al., 2009) leading to non-optimal functioning of individuals. The theory advocates that need thwarting will direct individuals to opt for substituting and often protective or self-defensive psychological adjustments that may prove costly for the health and welfare of individuals (Deci and Ryan, 2000). This has been empirically confirmed in multiple studies where thwarting of needs predicted various maladaptive outcomes (Bartholomew et al., 2011a). Other studies showed that need thwarting impaired work-related well-being (Bartholomew et al., 2014), and predicted ill-being (Chen et al., 2015). Moreover, psychological need thwarting predicted negative attitudes like turnover intention (Gillet et al., 2015), anger and bullying (Hein et al., 2015), etc., of employees. Along with that psychological need thwarting appeared to associate with maladaptive coping behaviors, e.g., binge eating (Schuler and Kuster, 2011; Verstuyf et al., 2013), self-injurious behaviors (Vansteenkiste et al., 2013), PWd (Shaheen et al., 2019) etc.

The environments that thwart individuals’ psychological needs allow them to develop maladaptive coping patterns (Vansteenkiste and Ryan, 2013). These findings are consistent with an important feature of BPNT, which states that people may use a variety of maladaptive coping ways when their psychological needs are thwarted (Deci and Ryan, 2000). To our knowledge, no previous study has yet attempted to examine the link between autonomy and relatedness need thwarting and PWd. PWd is a form of withdrawal behaviors which are known as maladaptive avoidance coping behaviors (see Polman et al., 2010). So based on empirical evidence given above, we propose that

*Hypothesis 2a.* Autonomy need thwarting is positively related to PWd.
*Hypothesis 2b.* Relatedness need thwarting is positively related to PWd.

### Thwarting of Psychological Needs as a Mediator Between CCB and PWd

Basic psychological needs theory lays on the premise that when psychological needs are frustrated, they explain the relationship between the damaging effects of the work environment and the ineffective functioning of an individual (Ryan and Deci, 2000). Numerous empirical studies have confirmed this principle. Bartholomew et al. (2014) concluded that thwarting of psychological needs mediated the relationship between perception of job pressure and burnout along with the competence need predicting somatic complaints. Thwarting of all three psychological needs explained the association of task variety, role conflict, and perceived leader support with affective commitment (Gillet et al., 2015).

Furthermore, the social environment and ill-being relationship were mediated by psychological need thwarting (Balaguer et al., 2012). Another study conducted by Gillet et al. (2012) revealed that psychological need thwarting explained the relationship between perceived organizational support and different facets of hedonic and eudaemonic work satisfaction (i.e., happiness and self-realization). Psychological need thwarting also served as an underlying mechanism between controlling behavior and various maladaptive outcomes like burnout, negative emotions, etc. (Bartholomew et al., 2011a).

To the best of our knowledge, no previous study has examined psychological need thwarting as an intervening mechanism in the relation between CCB and PWd. So the studies in which the relationship between controlling and maladaptive coping behaviors was mediated by psychological need thwarting (e.g., Olafsen et al., 2017; Bartholomew et al., 2018) have given us a hint to propose this mediating effect. It is because withdrawal behaviors are maladaptive avoidance coping behaviors (see Polman et al., 2010), and CCB is an involuntary behavior performed due to coercion, so it seems reasonable to explore psychological need thwarting as an intervening variable in our theoretical model. To add further, it is an essential characteristic of BPNT that when psychological needs are thwarted, people may adopt an array of maladaptive coping ways. Thus, based on the above discussion we propose that;

*Hypothesis 3a.* Autonomy need thwarting mediates the positive relation between CCB and PWd.
*Hypothesis 3b.* Relatedness need thwarting mediates the positive relation between CCB and PWd.

## Materials and Methods

### Sample and Procedure

Participants including clerks, administrative assistants, teaching assistants, personal assistants, research assistants, secretaries, office assistants, and receptionists, were approached through personal contacts, and then volunteers were given a paper and pencil based survey. The survey contained a cover letter along with an empty envelope. The cover letter elucidated the aim of the research, a guarantee of complete confidentiality of information, and instructions to return the complete survey in closed envelopes.

To select the sample size, a recommended criterion by Hair et al. (2010) was considered. They recommended researchers to engage 15 respondents against each scale item. Since there were 22 items in this study, so at least 330 respondents were mandatory to meet the criterion. However, this study had 368 respondents which accomplished this *a priori* condition.

Researchers received 385 filled questionnaires (approximately 77% response rate) out of 499 distributed surveys. After eradicating questionnaires with incomplete data, the remaining useable surveys were 375 (a useful response rate of 75%). Mahalanobis distance χ^2^ (7) = 24.32, *p* < 0.001 detected seven outliers which were excluded from further analysis. This left us with a final sample of 368 (approximately 74% final response rate).

Among 368 respondents, 247 were males, and 121 were females. Based on designation, the sample consisted of 162 clerks, 194 assistants, and 12 others. The mean age of respondents was (M*_*age*_* = 30.52 years, SD = 3.96), and they ranged from 23 to 46 years.

### Measures

We reversed the order of study measures as recommended by Podsakoff et al. (2003). This was done as a procedural remedy to decrease potential biasness resulting from item priming effect. Thus, we asked the dependent variable (i.e., PWd) questions first, autonomy and relatedness need thwarting second, and lastly, CCB questions (independent variable) followed by negative affectivity (NAf). As a result, employees’ rating of CCB is not likely to have any effect on other variables. A recall period of 2 months was used to have a considerable sample size. This is aligned with studies done in the past (e.g., Wang and Wang, 2017). The reliability of the scales was measured with Cronbach’s alpha coefficient (α) which is the most widely used objective measure of reliability (Tavakol and Dennick, 2011, p. 53).

#### CCB

It was measured with a five-item adopted scale originally developed by Vigoda-Gadot (2007). An example item is “I feel that I am forced to assist my supervisor against my will and beyond my formal job obligations.” Participants were requested to describe the frequency (1 = never, 5 = many times) of facing CCB events at the workplace within the previous 2 months. The scale showed Cronbach’s alpha of 0.88.

#### Psychological Need Thwarting

Psychological Need Thwarting Scale (PNTS; Bartholomew et al., 2011b) was adapted to measure employees’ view of the autonomy and relatedness need thwarting. Scale originally consisted of 8 items to measure autonomy and relatedness need thwarting (four items each) in a sports context. However, we used six items (3 items each) to measure these needs in the work context. The original PNTS underwent two modifications. First, the instruction was customized to be read as given: “considering your work environment during the last 2 months, please indicate how much you agree or disagree with statements given below.” Second, few items underwent modifications. An example item for this modification is “I feel prevented from making choices with regard to the way I engage in extra-role duties.” The two items excluded were “I feel under pressure to agree with the training regimen I am provided” (autonomy thwarting), and “I feel other people are envious when I achieve success” (relatedness thwarting), as they were irrelevant to the current study. Employees’ responses were made on a five-point Likert scale (1 = strongly disagree to 5 = strongly agree). An exploratory factor analysis yielded two factors, i.e., a subscale of autonomy (3 items, Cronbach’s alpha = 0.84), and another of relatedness need thwarting (3 items, Cronbach’s alpha = 0.89) consistent with previous findings (e.g., Bartholomew et al., 2011b).

#### PWd

We measured this construct by adopting an eight-item scale developed by Lehman and Simpson (1992). Another item (i.e., Showed effort to look busy even when not) was adapted from PWd scale for teachers developed by HajiGhasemi and Hasanzadeh (2013). It was added because it measured a PWd behavior that is often talked about in literature (e.g., Colquitt et al., 2011). Previous studies relied on self-report measurement of this construct (see Wang and Huang, 2019) due to its capacity of being easily overlooked by “significant others” (Sackett and DeVore, 2002). Hence, we also used a similar approach. The instruction was customized as given: During the last 2 months…; and then participants were requested to describe the frequency (1 = never, 5 = many times) of engaging in given thoughts or behaviors. To determine the homogeneity of the scale, exploratory factor analysis was performed. The analysis revealed that an item, i.e., “I left work station for unnecessary reasons” did not load on expected factor. So, it was not included in further analysis. The remaining eight items loaded on one factor with the Cronbach’s alpha coefficient of 0.89.

#### Control Variables

Previous studies have shown that gender (1 = Males, 2 = Females) and age (years) is related to withdrawal behaviors (e.g., Volpone and Avery, 2013; Mawritz et al., 2014). So they were controlled in this study. Likewise, we also controlled for dispositional NAf, which is described by Watson and Clark (1984) as a person’s natural tendency to experience distressing emotional states. Previous studies showed that persons with high NAf adopted such coping behaviors that were maladaptive (e.g., Nicolai et al., 2016). So to analyze the pure effect of CCB on PWd (maladaptive coping behavior), we controlled NAf. It was measured with three negative adjectives that Kim et al. (2015) adopted for their study from PANAS scale (Watson et al., 1988). Respondents answered the frequency (1 = Never, 5 = Always) of their general feelings in terms of given adjectives. The Cronbach’s alpha coefficient of the scale was 0.69.

### Data Analysis

To establish the reliability and validity of the study constructs, a confirmatory factor analysis (CFA) was performed through AMOS software. We probed and contrasted a five-factor model with three alternative models. To evaluate the risk for common method bias, we also made a comparison of the five-factor model and a common latent factor (CLF) model (Podsakoff et al., 2003).

Hu and Bentler (1999) suggested that to analyze the goodness of a model fit, following cutoff values should be used. A value close to 0.95 for both CFI and TLI and a value close to 0.08 and 0.06 for both SRMR and RMSEA, respectively, show good fit of a model. To test the proposed hypotheses regarding direct relationships among variables, Pearson’s bivariate correlation was used as a preliminary test. Then the PROCESS Macro for SPSS developed by Hayes (2013) was used as a robust test for testing these hypotheses. The same macro was used for testing the mediation. It gave us the opportunity to analyze the *total* indirect effect, and the *individual* indirect effects of both autonomy and relatedness need thwarting. It also allowed us to conduct contrast test of individual indirect effects for investigating the difference between them. Besides that, we calculated effect ratios to check the proportion of relationship of CCB with PWd that was explained by two mediators.

## Results

### Preliminary Analyses

Hair et al. (2010) recommended removing questionnaires having more than 50% missing entries. So ten questionnaires fell in this criterion and were excluded. After that, the data normality was assessed. The skewness and kurtosis values ranged from −0.97 to 0.20 and 0.02 to −1.09, respectively. These values were considerably lower than the suspicious values [i.e., ≥2.0 for skewness and ≥7.0 for kurtosis (Curran et al., 1996)].

### Measurement Model

Table 1 summarizes the results of CFA. The model with five latent constructs (i.e., CCB, autonomy thwarting, relatedness thwarting, PWd, and NAf) demonstrated a good fit to the data, [χ^2^ (199) = 307.33, *p* < 0.001; χ^2^/*df* = 1.54; CFI = 0.97; TLI = 0.97; SRMR = 0.041; RMSEA = 0.039] (Hu and Bentler, 1999). Next, the five-factor model was contrasted with a general factor model and another model in which items were allowed to load simultaneously on their respective latent factors along with a CLF, to assess the risk of common method bias (Podsakoff et al., 2003). The contrast showed a better fit of the five-factor model than the model with general factor (see Table 1). Moreover, only 5% variance was explained by common method factor. It was well below the recommended threshold of 25% (Williams et al., 1989). Additionally, we observed the Parsimony Normed Fit Index (PNFI) of five-factor model and compared it with PNFI of CLF model. The expected measurement model showed better PNFI = 0.80 in comparison to CLF model (PNFI = 0.72). Thus, we decided to conduct further analysis with scales specified in five-factor model.

**TABLE 1:** Comparison of fit of alternative models.

**Model**		**Latent factors**	**χ^2^(*df*)**	**χ^2^/df**	**CFI**	**TLI**	**RMSEA**	**SRMR**	**Model comparison**	**Δχ ^2^**	**Δ*df***
1	Measurement model	CCB, AutThw, RelThw, PWd, NAf	307.33^∗∗∗^	1.54	0.97	0.97	0.039	0.041			
			(199)								
2	One-factor model	General factor	2023.87^∗∗∗^	9.64	0.53	0.48	0.153	0.145	2 versus 1	1716.54^∗∗∗^	11
			(210)								
3	Four-factor model	CCB, Need thwarting, PWd, NAf	802.29^∗∗∗^	3.95	0.85	0.82	0.090	0.105	3 versus 1	494.96^∗∗∗^	4
			(203)								
4	Measurement model with common method factor	CCB, AutThw, RelThw, PWd, NAf, CMF	232.05^∗∗∗^	1.31	0.99	0.98	0.029	0.029	4 versus 1	75.28^∗∗∗^	22
			(177)								

*CMF, common method factor; NAf, negative affectivity; AutThw, autonomy thwarting; RelThw, relatedness thwarting; PWd, psychological withdrawal. ^∗∗∗^*p* < 0.001.*

### Descriptive Statistics

In Table 2, we summarized the means, standard deviations, Cronbach’s alpha, composite reliability, average variance extracted (AVE), and correlations (*r*) for all the variables of study. The Cronbach’s alpha and composite reliability were well above an acceptable threshold of 0.60 and 0.70, respectively (Hair et al., 2010). So this evidenced a good internal consistency and validity of the study constructs. Furthermore, we established convergent validity of variables by examining composite reliability and AVE values. These values were higher (except for NAf with AVE = 0.44) than the suggested cut-off of 0.70 and 0.50, respectively (Hair et al., 2010). But the lower value of NAf was still acceptable since its CR reached a recommended threshold of 0.70 as per Fornell and Larcker (1981) criterion. Another conservative criterion of Fornell and Larcker (1981) was used for establishing the discriminant validity of constructs. As per this criterion, the square root of AVE of all measures should exceed their correlations with other constructs. The results showed that the square roots of AVE of all constructs were greater than their correlations with other constructs as reported in Table 2, so discriminant validity was also established.

**TABLE 2:** Means, standard deviations, reliabilities, and correlations.

		**M**	**SD**	**α**	**CR**	**AVE**	**1**	**2**	**3**	**4**	**5**	**6**	**7**
1	Gender	−	−	na	na	na	—						
2	Age	30.53	3.95	na	na	na	–0.08	—					
3	Negative Affectivity	3.27	0.89	0.69	0.70	0.44	0.02	0.02	(0.66)				
4	Compulsory Citizenship Behavior	3.30	0.87	0.88	0.88	0.60	–0.02	–0.03	0.26^∗∗^	(0.77)			
5	Autonomy Thwarting	3.11	0.82	0.84	0.85	0.66	0.04	–0.13	0.24^∗∗^	0.39^∗∗^	(0.81)		
6	Relatedness Thwarting	3.85	0.91	0.89	0.89	0.74	0.02	–0.05	0.26^∗∗^	0.30^∗∗^	0.32^∗∗^	(0.86)	
7	Psychological Withdrawal	3.37	0.79	0.89	0.89	0.50	0.11^∗^	0.15^∗∗^	0.20^∗∗^	0.41^∗∗^	0.51^∗∗^	0.35^∗∗^	(0.71)

**N* = 368. na, not applicable; CR, composite reliability; AVE, average variance extracted. Diagonal represents the square root of AVE; while below the diagonal the estimated correlations are represented. ^∗^*p* < 0.05, ^∗∗^*p* < 0.01.*

The correlation results among study variables confirmed that as anticipated, CCB related positively with PWd (*r* = 0.41, *p* < 0.01), thwarting of autonomy (*r* = 0.39, *p* < 0.01) and relatedness (*r* = 0.30, *p* < 0.01) need. Moreover, PWd showed a positive correlation with thwarting of both autonomy (*r* = 0.51, *p* < 0.01) and relatedness (*r* = 0.35, *p* < 0.01) need. These results offered preliminary support for our hypotheses.

### Test of the Hypotheses

**FIGURE 1:**
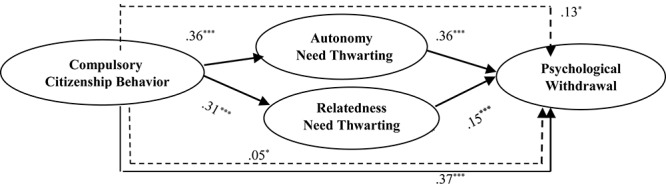
Results of the hypothesized research model. Dashed lines show indirect paths for autonomy (H_3a_) and relatedness (H_3b_). *N* = 368, ^∗^*p* < 0.05, ^∗∗∗^*p* < 0.001.

The results of multiple mediation analysis for hypotheses testing are summarized in Table 3 without covariates. First, CCB significantly related to PWd (*b* = 0.37, *SE* = 0.04, *p* < 0.001). Second, CCB was positively related to thwarting of autonomy (*b* = 0.36, *SE* = 0.04, *p* < 0.001) and relatedness (*b* = 0.31, *SE* = 0.05, *p* < 0.001) need. Thus, Hypothesis 1a and 1b were supported. Third, thwarting of autonomy need not only significantly related to PWd (*b* = 0.36, *SE* = 0.05, *p* < 0.001; Hypothesis 2a supported), but also yielded significant indirect effect (effect = 0.13, 95% CI [0.09,0.19]) of CCB. Hence, hypothesis 3a was supported. Similarly, thwarting of relatedness need (*b* = 0.15, *SE* = 0.04, *p* < 0.001) was significantly related to PWd (Hypothesis 2b accepted). Moreover, thwarting of relatedness need also yielded significant indirect effect (effect = 0.05, 95% CI [0.02,0.09]) of CCB. Thus, we failed to reject hypothesis 3b. There was partial mediation since the direct effect i.e., Ć path (*b* = 0.19, *SE* = 0.04, *p* < 0.001) remained significant. The test of differences confirmed that specific indirect effects of thwarting of both needs significantly differed from each other (*b* = 0.09, *SE* = 0.03, 95% CI [0.03,0.14]). The total amount of variance accounted for by the overall model, which included autonomy need thwarting, and relatedness need thwarting as mediators, was 34%. The hypotheses results are depicted in Figure 1.

**TABLE 3:** Results of the analyses for multiple mediation (without covariates).

**Psychological withdrawal**	**Coefficient**	**SE**	**Bootstrap 95% CI**	**Effect ratio**
*IV to mediators (A paths)*				
Compulsory Citizenship Behavior→Autonomy Thwarting	0.36^∗∗∗^	0.04		
Compulsory Citizenship Behavior→Relatedness Thwarting	0.31^∗∗∗^	0.05		
*Mediators to DV (B paths)*				
Autonomy Thwarting→Psychological Withdrawal	0.36^∗∗∗^	0.05		
Relatedness Thwarting→Psychological Withdrawal	0.15^∗∗∗^	0.04		
*Total effect of IV on DV (C path)*	0.37^∗∗∗^	0.04		
*Direct effect of IV on DV (Ć path)*	0.19^∗∗∗^	0.04		
Model *R*^2^	0.34			
*Total indirect effect of IV on DV through proposed mediators*	0.18	0.03	[0.13,0.24]	0.48
Compulsory Citizenship Behavior→Autonomy Thwarting→Psychological Withdrawal	0.13	0.02	[0.09,0.19]	0.36
Compulsory Citizenship Behavior→Relatedness Thwarting→Psychological Withdrawal	0.05	0.02	[0.02,0.09]	0.12
*Autonomy thwarting vs. Relatedness thwarting*	0.09	0.03	[0.03,0.14]	

**N* = 368. ^∗∗∗^*p* < 0.001.*

To rule out the possible alternative justification for the earlier results, the analysis was rerun with related control variables. However, the results showed that significant interrelationships between the variables remained unchanged. Thus, we reported results in Table 3 without covariates for the sake of parsimony and ease of comparison with previous research.

## Discussion

In the current study, we examined the relationship of CCB with psychological need thwarting. The study hypothesized that CCB is positively related to thwarting of autonomy and relatedness need. The results showed a significant positive relationship of CCB with thwarting of both autonomy and relatedness need. These results are in accordance with studies done in the past where pressurizing and controlling treatments from powerful, significantly thwarted individuals’ psychological needs (e.g., Hein et al., 2015; Liu et al., 2017; Bartholomew et al., 2018). The results are also aligned with studies where work stressors significantly thwarted employee psychological needs (e.g., Trepanier et al., 2016; Bartholomew et al., 2017). These results suggest that employees, who engage in citizenship behavior involuntary due to coercive treatment from “significant others,” might construe this unwanted pressure as a neglect of their innate need for autonomy. Along with that, this involuntary citizenship behavior due to undesired pressure from social partners breaks the social tradition of support and care from them. This might thwart employees’ psychological relatedness need.

The study further investigated psychological need thwarting as an antecedent to coping behavior of PWd by hypothesizing that autonomy and relatedness need thwarting are positively related to PWd. The results showed that both psychological needs significantly related to maladaptive coping behavior of PWd. These results are in accordance with studies done in the past, where need thwarting significantly related to maladaptive coping behaviors (e.g., Schultz et al., 2015; Olafsen et al., 2017). These results suggest that when employees’ need for autonomy and relatedness are thwarted by significant others, they engage in PWd to feel better and reciprocate to the treatment received.

Lastly, the study tested whether thwarting of psychological need serves as a mediational mechanism through which CCB stressor relates to employee PWd behavior. For this purpose the study hypothesized that autonomy and relatedness need thwarting mediates the positive relation between CCB and PWd relationship. The results revealed that thwarting of both needs mediated the positive relationship between CCB and PWd as outlined in BPNT (Ryan and Deci, 2000). These results are somewhat in accordance with studies done in past where psychological need thwarting significantly served as a mediator in explaining the relationship between work stressors and maladaptive coping behaviors (e.g., Gillet et al., 2015; Hein et al., 2015; Olafsen et al., 2017). Such results suggest that although both needs are conceptually distinguishable, their thwarting produces similar results that are negative in nature as recommended by Deci and Ryan (2000). So those who work at a lower level of hierarchy retaliate in similar way to thwarting of both needs and engage in PWd to emotionally cope with the frustration without being noticed.

We also found that although both autonomy and relatedness need thwarting served as mediators, particularly autonomy thwarting played a pivotal role in explaining positive CCB and PWd association.

### Theoretical Implications

The current research has some significant theoretical implications. First, by empirically establishing the link of CCB with psychological need thwarting, we have answered to a call made by Yam et al. (2014) regarding exploring the impact of CCB on more employee attitudes. Second, the study has also contributed to the literature on PWd by exploring psychological need thwarting as its new determinant. Third, to our knowledge, it is the first study to investigate the negative side of CCB from a motivational perspective and the first to examine thwarting of autonomy and relatedness needs as powerful employee motives to engage in PWd. The findings have added to the CCB literature and BPNT (Ryan and Deci, 2000). As autonomy thwarting appeared to be the most vital factor in explaining the association between CCB and PWd, it indicates that coercion, lack of choice is one of the most detrimental job characteristics as it thwarts one of the most fundamental needs, i.e., autonomy (Sheldon et al., 2001). These results are consistent with previous findings (e.g., Blanchard et al., 2009), and contribute toward the debate in SDT that some needs might be more germane in specific processes than others (see Greguras and Diefendorff, 2009; Vanhee et al., 2018). Moreover, thwarting of relatedness need also played a part in the positive relationship between CCB and PWd. This also added to the BPNT literature by confirming that involuntary citizenship behavior due to excessive coercion by seniors can undermine a junior’s sense of both autonomy and relatedness need (Barber, 2002) and enforce them to engage in maladaptive coping behaviors (Jang et al., 2016; Bartholomew et al., 2018).

### Practical Implications

The present research has few considerable practical implications. Previously, some literature indicated the role of pressure as a constructive and motivating force that is frequently necessary for work (e.g., Andrews and Farris, 1972). But our finding in the context of pressure to engage in CCB contradicts such literature. A deeper insight into the motivational mechanism pinpointing the employees’ maladaptive and compromised functioning, as a reaction to CCB, is crucial for the effective implementation of interventions that could reduce this malfunctioning. A logical way to prevent employees from coping with CCB by psychologically withdrawing is to address the satisfaction of their psychological needs. It seems reasonable that management should introduce alternative strategies to satisfy the psychological needs of employees. This could be done by investing in autonomy support (Liu and Fu, 2011), e.g., instead of forcing employees to do some extra-role duties, managers should communicate them the reason why they are being asked to perform these duties. Since, Bauer (2013) speculated that those employees are more vulnerable to future requests of OCB, who previously complied with manager’s request and such employees might get stuck in compulsory OCB spiral (Bauer, 2013); therefore, instead of forcing such employees, managers should take them into confidence and should discuss with them the circumstances and provide them with the opportunity to decide on their own. This might help employees to feel being cared for (relatedness need satisfaction) and autonomous (autonomy need satisfaction) and ultimately reducing their PWd. Additionally, the present findings highlight that it is crucial to train managers in practicing more autonomy and relatedness supportive behaviors for employees and less use of coercive treatment.

### Limitations and Future Research

The current study has a few limitations that are required to be accepted. First, due to the design of the study that was cross-sectional, we were unable to make inferences regarding causal relationships. So, the researchers in the future should attempt to re-examine this model longitudinally. A second limitation is a reliance on self-report measures of all study variables. Future researchers could use multi-source research design. A third limitation is that since data were collected from the clerical staff only, so the generalizability of the study is of concern. As thwarting of psychological need is an innate and basic mechanism (Deci and Ryan, 2000), we would anticipate it to account for the effects of CCB on employees of each level within the hierarchy. Therefore, to gain additional support for the generalizability of our results, studies in the future on similar topics should pay attention to employees working in other occupations and different levels of hierarchy. A fourth limitation is that the current study investigated thwarting of psychological needs as a motivational mechanism explaining CCB and PWd relationship. Future researchers may examine other cognitive and emotional intervening mechanisms in explaining this association. Finally, potential moderating variables from other theories and also within SDT, e.g., trait measure of autonomy need strength may also be examined. Recent research has highlighted its moderating role (e.g., Chen et al., 2015; Van Yperen et al., 2016).

## Conclusion

To conclude, the present study has added to the BPNT by indicated the thwarting of both autonomy and relatedness needs as motivational underlying mechanisms in explaining the positive association between CCB and PWd. In doing so, the study highlights the significance of the extent to which clerical workers perceive their basic psychological needs to be satisfied or thwarted. The study findings that autonomy need thwarting appeared to be the most vital factor in explaining the association between CCB and PWd contributes toward the discussion in SDT that some needs might be more relevant to specific processes than others.

## Data Availability Statement

The datasets generated for this study are available on request to the corresponding author.

## Ethics Statement

The protocol was reviewed and approved by the ethics committee of Government College University Faisalabad, Pakistan. The participants provided their written informed consent to participate in this study.

## Author Contributions

MB, KS, SS, MA, and ZJ: definition of research objectives, models, hypotheses and principal manuscript crafting. MB, KS, and SS: the provision of materials (i.e., questionnaires). MB and KS: data collection. KS, SS, MA, and MKB: data analysis plan and data analysis. MKB and ZJ: manuscript revision and proofreading. MB, KS, SS, MKB, MA, and ZJ: final approval.

## Conflict of Interest

The authors declare that the research was conducted in the absence of any commercial or financial relationships that could be construed as a potential conflict of interest.
